# Structural Connectivity-Based Parcellation of the Dopaminergic Midbrain in Healthy Subjects and Schizophrenic Patients

**DOI:** 10.3390/medicina56120686

**Published:** 2020-12-10

**Authors:** Gianpaolo Antonio Basile, Alessia Bramanti, Salvatore Bertino, Giuseppina Cutroneo, Antonio Bruno, Adriana Tisano, Giuseppe Paladina, Demetrio Milardi, Giuseppe Anastasi

**Affiliations:** 1 Brain Mapping Lab, Department of Biomedical, Dental Sciences and Morphological and Functional Images, University of Messina, 98124 Messina, Italy; bertinosalvatore404@gmail.com (S.B.); gcutroneo1@gmail.com (G.C.); dptbiosciences@gmail.com (G.A.); 2 IRCCS Centro Neurolesi “Bonino Pulejo”, 98124 Messina, Italy; alebramanti@gmail.com (A.B.); peppepaladina@hotmail.it (G.P.); 3 Psychiatry Unit, Department of Biomedical and Dental Sciences and Morphological and Functional Imaging, University of Messina, 98124 Messina, Italy; abruno@gmail.com; 4 Physical, Rehabilitation Medicine and Sport Medicine Unit, University Hospital G. Martino, 98124 Messina, Italy; atisano@libero.it

**Keywords:** schizophrenia, midbrain, substantia nigra, diffusion weighted imaging tractography, ventral tegmental area, neuroimaging

## Abstract

*Background and objectives*: Functional deregulation of dopaminergic midbrain regions is a core feature of schizophrenia pathophysiology. Anatomical research on primates suggests that these regions may be subdivided into distinct, topographically organized functional territories according to their connectivity to the striatum. The aim of the present work was the reconstruction of dopaminergic midbrain subregions in healthy subjects and schizophrenic patients and the evaluation of their structural connectivity profiles. *Materials and Methods*: A hypothesis-driven connectivity-based parcellation derived from diffusion tractography was applied on 24 healthy subjects and 30 schizophrenic patients to identify distinct territories within the human dopaminergic midbrain in vivo and non-invasively. *Results*: We identified a tripartite subdivision of dopaminergic midbrain, including limbic, prefrontal and sensorimotor territories. No significant differences in structural features or connectivity were found between subjects and patients. *Conclusions*: The parcellation scheme proposed herein may help to achieve detailed characterization of structural and functional anomalies of the dopaminergic midbrain in schizophrenic patients.

## 1. Introduction

Schizophrenia (SZ) is a common, invalidating mental disease with a huge social and economical burden, with still unknown etiology and pathogenesis [1]. Among biological factors, deregulation of the dopaminergic system has been long-time implicated in the pathophysiology of the most common symptoms [2]. In light of the central role played by dopamine in brain systems regulating salience detection and attribution, it has been suggested that positive symptoms, such as delusions or hallucinations, could emerge as consequence of an aberrant attribution of salience to otherwise irrelevant elements of every-day life [3,4,5]. On the other hand, the effects of altered dopaminergic neurotransmission in terms of value representation, reward processing and reinforcement learning may underlie motivation-related negative symptoms such as anhedonia or abulia [6].

Substantia nigra pars compacta (SNc) and the nearby adjacent ventral tegmental area (VTA) are the most important dopaminergic brain regions implied in schizophrenia [7,8,9,10]. While traditionally these regions are often regarded as clearly distinct in functional terms, being SNc mostly implied in motor control [11,12] and VTA in salience and reward processing [13,14], a growing line of research suggests that these regions can be considered as being part of a unified “midbrain dopaminergic complex” [15,16,17] with topographically organized, cytoarchitectonically distinct functional territories. Studies in primate models demonstrated that midbrain dopaminergic neurons can be subdivided in three tiers according to their cytoarchitecture, connectivity and immunoreactivity pattern: (i) a ventro-lateral, calbindine-negative tier mostly connected to motor striatum; (ii) a densocellular, calbindine-negative, intermediate tier mostly connected to associative striatum; (iii) a dorso-medial, sparse-cells, calbindine-positive tier that is connected to limbic territories of dorsal striatum, ventral striatum and cortex [18,19,20,21,22,23].

It can be hypothesized that these different functional compartments within the human SNc/VTA may show selective functional or structural alterations in schizophrenic patients. However, to the best of our knowledge, only a few attempts have been made for differentiating distinct sub-territories of the human midbrain dopaminergic complex [24,25] and their role both in the healthy human brain [26,27] and in schizophrenic patients [28] is still poorly understood.

In the present work, we employed structural and diffusion-weighted magnetic resonance imaging datasets from 24 healthy controls (HC) and 30 schizophrenic patients (SZ) from the Mind Clinical Imaging Consortium repository (*MCICShare*) [29,30,31] in order to evaluate topographically selective differences in structural connectivity of the dopaminergic midbrain complex. We adopt a two-step hypothesis-driven connectivity-based parcellation approach [32,33,34] based on multi-tissue constrained-spherical deconvolution (CSD) tractography [35,36] to reconstruct distinct, topographically arranged territories within the human SNc/VTA according to midbrain-striatal connectivity. Then, we compared volumetric and structural connectivity differences within these territories between HCs and SZ patients.

## 2. Materials and Methods

### 2.1. Patients and Controls Selection

Data used in the preparation of this work were obtained from the Mind Clinical Imaging Consortium database through the Mind Research Network (www.mrn.org). The MCIC project was supported by the Department of Energy under Award Number DE-FG02-08ER64581. MCIC is the result of efforts of co-investigators from University of Iowa, University of Minnesota, University of New Mexico, Massachusetts General Hospital.

Dataset from 24 HCs and 30 SZ patients have been collected from the *MCICShare* repository. The two groups were matched for age (range 18–60 years) and sex (HC: 32 years± 11, 14 males, 10 females; SZ: 32 years ± 10, 22 males, 8 females). Inclusion criteria for the patient group was based on diagnostic criteria for schizophrenia, schizoaffective or schizophreniform disorders as defined in the fourth edition of Diagnostic and Statistic Manual for Mental Disorders (DSM-IV). The control group was composed of subjects with no history of actual or previous psychiatric disorders or substance dependence, including having received symptomatic treatment with antidepressant, anxiolytic or hypnotic drugs for more than 2 weeks and less than 6 months after the study.

Common exclusion criteria for patients and controls were: IQ lower than 70, as measured with a standardized test; clinical history of head trauma with prolonged loss of consciousness, neurosurgical intervention, neurological disease, severe or disabling diseases; contraindications to MRI scan such as gravedance or metal implants.

Structural clinical interview for DSM-IV (SCID/SCID-NP for controls) and Comprensive Assessment of Symptoms and History (CASH) [37] have been used to evaluate clinical history and psychiatric symptoms in patients and controls. In patients, severity of positive and negative symptoms has been scored using the Scale for Assessment of Positive Symptoms (SAPS) and Scale for Assessment of Negative Symptoms (SANS), respectively [38,39]. Only one patient is neuroleptic-naive. Extrapyramidal symptoms have been assessed using the Simpson and Angus Scale (SAS) [40,41]. Demographic and clinical data related to patients and controls are resumed in Table 1.

### 2.2. Data Acquisition

Since data were col1ected from a multi-site study repository, only datasets acquired in the acquisition site C were considered, to ensure uniformity among technical and acquisition features. All data were collected on a 3T MRI scanner (Siemens Trio, Erlangen, Germany) with the following parameters: for T1-weighted structural scans, TR = 2530 ms, TE = 3.79 ms, FA = 7, bandwidth = 181, voxel size 0.625 × 0.625 × 1.5 mm; FOV matrix: 256 × 256 × 128 mm; FOV = 16 cm (extended to 18 cm for whole brain coverage); for diffusion weighted imaging (DWI) scans, TR = 10,500 ms, TE = 98 ms, b-values 0 and 1000 s/mm^2^, voxel size 2 × 2 × 2 mm, NEX = 2, band width = 1342, 64 slices, 12 directions [31].

### 2.3. Data Preprocessing

As a first step, T1 weighted images have been resampled to an isotropic voxel size of 1 × 1 × 1 mm using linear interpolation. DWI images underwent denoising and Gibbs ringing artifacts removal [42,43] motion and Eddy Currents correction using the EDDY pipeline included in FMRIB Software Library (FSL) software [44]. Since no reverse phase encoding or fieldmap acquisition were available, geometric and susceptibility-weighted distortions were corrected by non-linear registration between B0 DWI image and T1-weighted structural image [45,46] using a mutual information inter-modal cost function [47]. This step has been implemented using Niftyreg [48]. The obtained transform was then applied to all DWI volumes with automatic gradient reorientation using the mrtransform utility featured in the MRtrix3 software [49]. Finally, DWI volumes underwent B1-field inhomogeneity correction [50]. All data were quality-checked by visually inspecting the outputs of each preprocessing step, in particular to ensure the accuracy of non-linear registration.

### 2.4. Post-Processing

T1-weighted images were skull-stripped [51] and underwent cortical and subcortical segmentation using FAST and FLIRT [50,52]; structural volumes were then non-linearly coregistered to Montreal Neurological Institute (MNI-152) standard brain template (1 mm^3^ resolution version) using FLIRT and FNIRT utilities on the FSL software [53] and direct and inverse transformations were saved.

Fractional anisotropy (FA) maps were obtained from preprocessed DWI volumes after tensor fitting and FA estimation using the commands dwi2tensor and tensor2metric featured in MRtrix3 software [54,55,56]. The CSD signal modeling was implemented by extracting a multi-tissue unsupervised response function directly from DWI single-shell scans [57].

Fiber orientation distribution (FOD) functions were then estimated using a super-resolved multi-tissue approach setting maximum spherical harmonic degree (lmax) to 8, to take advantage of the hard non-negativity constraint of CSD when dealing with non-high-angular-resolution diffusion imaging (HARDI) data [36,58].

### 2.5. Regions of Interest (ROI) Selection

Cortical and subcortical regions of interest were extracted by non-linear registration to each subject’s space of two standard-space atlases: Automated Atlas Labeling-v3 (AAL3) [59] for cortical parcellation and CIT68 Reinforcement Learning Atlas [60] for subcortical parcellation. The MNI-registered versions of these atlases were non-linearly coregistered to each subject’s space using previously obtained inverse transformations between T1 scans and the MNI template. From subcortical parcellation, ROIs for caudate, putamen and nucleus accumbens on each side were merged to obtain a unified striatal seed ROI. For connectivity-based parcellation, cortical areas were merged into three target ROIs for each hemisphere: (i) a limbic group including orbitofrontal cortex, frontal pole, cingulate cortex and parahippocampal gyrus; (ii) a prefrontal group including the remaining dorsomedial, dorsolateral, ventromedial and ventrolateral prefrontal cortex; (iii) a sensorimotor group including supplementary motor area, precentral gyrus, postcentral gyrus and paracentral lobule. Since the DWI dataset has been previously non-linearly registered to T1 scans, each of these ROIs was checked for accurate localization on DWI volumes and, if needed, manually corrected by a trained neuroanatomist.

**Figure 1:**
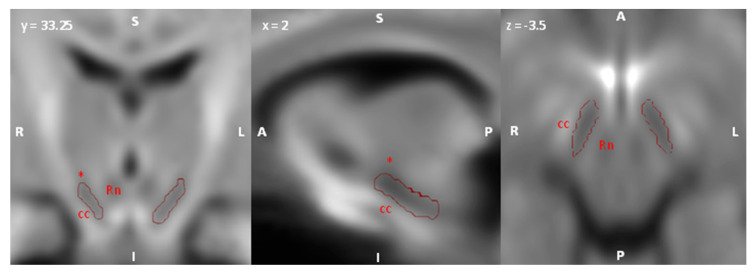
Identification of the Substantia nigra pars compacta (SNc)/ventral tegmental area (VTA) complex on study-specific fiber orientation distribution (FOD) template. Axial, sagittal and coronal sections showing the putative boundaries of SNc/VTA complex. SNc/VTA region was defined as an area of marked hypointensity situated anteriorly to the red nucleus (Rn), posteriorly to the crus cerebri (cc) and inferiorly to the subthalamic nucleus (*). S: superior; I; inferior; R: right; L: left; A: anterior; P: posterior.

For what concerns ROIs of the dopaminergic SNc/VTA complex, since the correction of susceptibility-weighted artifacts based on registration to T1-weighted scans was not sufficient to ensure a correct registration of midbrain regions, that are usually more affected by such artifacts [44], we adopted a manual delineation strategy involving custom, study-specific FOD templates. FOD maps were intensity normalized and a study-specific template was obtained by iterating a symmetric, unbiased FOD registration algorithm which includes FOD reorientation using apodised delta functions [61,62,63] using the dedicated MrTrix3 commands. To account for possible differences between patients and controls, two population templates were obtained from healthy subjects and schizophrenic patients, respectively. The dopaminergic midbrain complex was identified as a region of hypointensity located in the ventral midbrain anteriorly to the red nucleus, posteriorly to the crus cerebri and inferior to the subthalamic nucleus (Figure 1).

The obtained SNc/VTA ROIs were then transformed back to each subject’s native space, visually checked for correct localization on B0 and FOD images and manually corrected if needed.

### 2.6. Connectivity-Based Parcellation and Tractography

To parcellate an SNc/VTA ROI according to its striatal connectivity, a two-step hypothesis driven parcellation approach was implemented [64]: as a first step, striatum was subdivided into limbic, prefrontal and sensorimotor territories according to cortico-striatal connectivity; then, connectivity between these functional territories and the SNc/VTA ROIs was reconstructed and a second connectivity based-parcellation was performed on SNc/VTA ROIs. The common pipeline used for connectivity-based parcellation involved:

1. Seed-based tractography: 5000 streamlines were reconstructed seeding from seed ROIs (striatum, SNc/VTA) to the ipsilateral target ROIs (cortical groups) with the following parameters: algorithm IFod2 [65] step size 1.25, maximum angle 30. Target ROIs were used as inclusion mask; streamlines reaching each of the target masks were counted as connecting the source voxel to that target, and then immediately terminated. No exclusion masks were employed.
2. Tractogram-to-voxel mapping: by applying the track-density-imaging (TDI) framework, each tractogram was mapped to an image in which intensity is defined as the number of streamlines passing through a given grid element [66], corresponding in dimension and voxel size to seed ROI; track-density streamline maps were then multiplied to binarized seed ROIs to obtain tractograms endpoint distribution.
3. Classification: each map obtained from the previous step was normalized by dividing each voxel’s intensity to the mean intensity of the map, in order to obtain comparable intensity values: then, each voxel in the seed ROI was classified using a hard segmentation algorithm (find_the_biggest command on FSL) that assigns it to the map showing higher intensity.
4. Maximum probability maps reconstruction: for visualization purposes, each individual map was non-linearly coregistered to the corresponding study-specific FOD templates, binarized and summed up across the whole group to obtain maximum probability maps of each parcel at the group level. A threshold of 50% percent was assigned to show only voxels overlapping in at least half of the sample.

### 2.7. Quantitative Analysis

To evaluate structural features of SNc/VTA and striatal parcellation in HC vs. SZ, different quantitative measures were estimated. Streamline density index (SDI) [32,67,68,69] was obtained from volumes of each striatal and SNc/VTA parcel at subject-level using the following formula:

(1) $$SDI=\frac{v_{parcel}}{V_{seed}}\;\times \;100$$

where *v_parcel_* is the volume of each connectivity-based parcel and *V_seed_* the volume of the corresponding seed ROI.

In addition, we sampled mean FA values along 10,000 streamlines randomly seeded from each SNc/VTA parcel (parameters: algorithm IFod2, step size 1.25, maximum angle 30). To evaluate group-level differences, each of these quantitative estimates (SDI from SNc/VTA parcellation and FA from SNc/VTA parcel connectivity) was fitted into a general linear model (GLM) with a three-way ANOVA design, setting side and parcel type (Limbic, Prefrontal and Sensorimotor) and diagnosis (HC or SZ) as independent variables. Post-hoc tests were adjusted for multiple comparisons using Bonferroni’s test (significant α level < 0.05). All statistical analysis was conducted using IBM SPSS Statistics, v25 (IBM Corporation, Armonk, NY, USA).

## 3. Results

**Figure 2:**
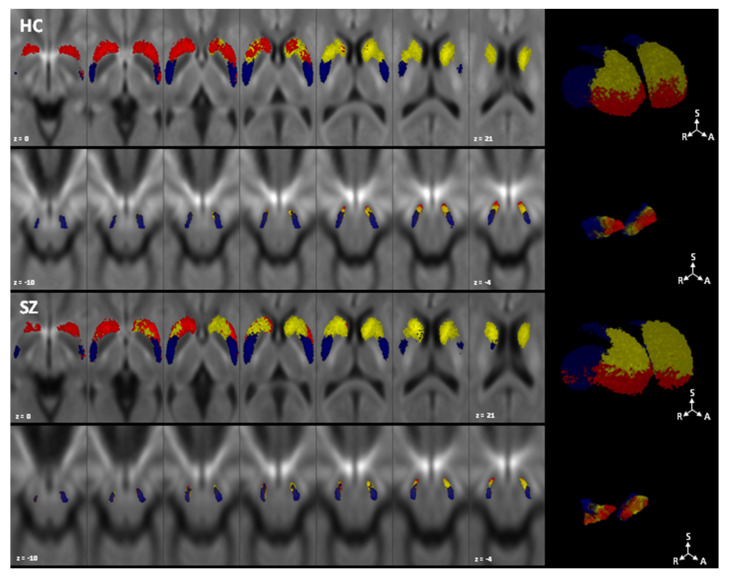
Topographical organization of SNc/VTA complex (lower row) according to striatal connectivity-based parcellation (upper row). Maximum probability maps showing only voxels overlapping in at least half of the sample are overlaid on study-specific FOD templates. Healthy controls are shown in the first two rows, and schizophrenic patients in the latter. Parcels mostly connected to the limbic cortex are colored in red, parcels with higher connectivity to prefrontal cortex are shown in yellow and parcels with higher connectivity to sensorimotor cortex are depicted in blue. 2D images are shown in radiological convention. HC: healthy controls; SZ: schizophrenic patients; S: superior; R: right; A: anterior.

The results from connectivity-based parcellation of striatum and SNc/VTA are depicted in Figure 2.

For both striatal and SNc/VTA parcellation, all parcels were obtained in 100% of subjects, and all parcels showed at least one voxel overlapping between subjects in at least 50% of the sample (12/24 for HC, 15/30 for SZ). Striatal parcellation is arranged with a ventro-dorsal and antero-posterior gradient, with the limbic cluster occupying mainly the nucleus accumbens and the most ventral part of caudate and putamen, the prefrontal cluster located in the dorsal anterior part of the caudate and putamen and the sensorimotor cluster that spreads over the posterior portion of putamen and to a lesser extent to the dorsal portion of caudate. In comparison, SNc/VTA parcellation shows a medio-lateral and antero-posterior topographical arrangement, with an anterior and dorsal-limbic territory, an intermediate and ventral prefrontal territory and a posterolateral sensorimotor territory.

Table 2 shows the mean values for striatal and SNc/VTA SDI and FA from connectivity analysis. For striatal parcellation, three-way ANOVA revealed significant effects of side (F(1,312) = 164.761, *p* < 0.01, η^2^ = 0.346), parcel (F(2,312) = 126.359, *p* < 0.01, η^2^ = 0.448) and side × parcel interactions (F(2,312) = 101.646, *p* < 0.01, η^2^ = 0.95). Post-hoc analysis for parcel resulted in significant differences between both sensorimotor and limbic (mean difference = 3.52, *p* < 0.01), limbic and prefrontal (mean difference = 2.92, *p* < 0.01) and prefrontal and sensorimotor parcels (mean difference = 6.54; *p* < 0.01). In addition, striatal parcellation reported significative SDI differences between the left and right side for most of parcels in both controls and patients with higher SDI values in the right sides for limbic clusters, (mean difference = 7.872, *p* < 0.01) and for prefrontal clusters (mean difference = 7.508, *p* < 0.01), while higher SDI values are in the left side for sensorimotor clusters (mean difference = 2.456, *p* < 0.01); no significant effects were found for diagnosis and its possible interactions.

SNc/VTA parcellation showed also a significant effect of parcel type (F(2,312) = 84.485, *p* < 0.01, η^2^ = 0.351) and side×parcel interactions (F(2,312) = 5.232, *p* < 0.01, η^2^ = 0.032). Post-hoc analysis for parcel revealed significant differences between limbic and sensorimotor clusters (mean difference = 16.43, *p* < 0.01) and prefrontal and sensorimotor clusters (mean difference = 14.28, *p* < 0.01), but not between limbic and prefrontal parcels. In particular, limbic cluster have significantly higher values on the right side (mean difference = 4.125, *p* = 0.037), while sensorimotor clusters show significantly higher values on the left side (mean difference = 4.73, *p* = 0.017). Again, no significant effects were found for diagnosis and related interactions.

For FA analysis, three-way ANOVA revealed no significant main effects of side and parcel, but a trend toward significance was found for the main effect of diagnosis (F = 8.116; *p* = 0.05), with SZ patients exhibiting slightly lower FA values (mean difference = 0.036). No significant interactions with side or parcels were found.

## 4. Discussion

In the present study we demonstrate that, in both HC and SZ patients, a similar topographical organization of connectivity between cerebral cortex, striatum and dopaminergic midbrain regions can be identified. In line with primate models of striatal organization [70,71] and with other neuroimaging investigations in the human brain [26,72], we found a ventral vs. dorsal topographical organization of cortico-striatal connectivity, with ventral striatal regions mostly connected to limbic cortical targets such as orbitofrontal and anterior cingulate cortex. The anterodorsal caudate and putamen are mostly connected to associative prefrontal regions, while sensorimotor cortical connectivity preferentially involves the dorsal caudate and putamen. We also show that a similar topographical organization is reflected in midbrain-striatal connectivity patterns, that show a medio-lateral topography with medial portions mostly connected to limbic striatum and lateral portions mostly connected to sensorimotor striatum, while associative striatum occupies an intermediate portion. To our knowledge, just a few neuroimaging studies of the human brain assessed the topographic organization of midbrain dopaminergic regions. An earlier investigation based on diffusion imaging [24] found a similar medio-lateral organization of connectivity, with medial SN mainly connected to ventral striatum and lateral SN mostly connected to the dorsal striatum. A recent investigation applied an unsupervised clustering algorithm to whole brain connectivity of SN and found three clusters with similar topographical organization [25]. In the current work, we extend the analysis to the SNc/VTA region of interest as defined by a recent multi-contrast MRI atlas [60]; plus, we opted for a two-step hypothesis-driven parcellation approach, in order to directly test the hypothesis about topographical organization derived from primate anatomical studies. The above described connectivity patterns partially reflect the functional organization of dopaminergic striatal circuitry described in primate models [19,21,22,23,73], although a clearly-described ventro-dorsal organization pattern could not be identified. While this could be in part conditioned by all the intrinsic limitations of tractography, that is not able to identify neuronal terminations at the synaptic level [74,75], the good agreement with animal findings support the hypothesis of a tripartite SNc/VTA subdivision in the human brain. These distinct topographical regions may play a crucial role in integrating different aspects of behavioral response to salient stimuli, from salience detection to finalized motor responses, incentive learning and habit formation [20].

In addition, our results suggest that such topographical organization could be maintained without relevant structural alterations in SZ patients, as we failed to find significative differences in SDI of both striatal and midbrain parcellation between patients and controls. In SZ patients, alterations in the functional activation of SNc/VTA in response to salient stimuli is well-documented from different functional MRI investigations [7,76,77]. It has been suggested that alteration in structural connectivity of the dopaminergic midbrain, in particular between SNc/VTA and prefrontal cortical regions, could explain these functional abnormalities [8,9,10,78]. To test this hypothesis, we extracted mean FA, that is commonly used as a surrogate measure of white matter structural integrity in SZ patients [79,80,81], from a sample of tractograms randomly seeded from each midbrain parcel. While we found slightly decreased FA values along midbrain-related white matter pathways, in line with recent research [82], we were not able to identify topographically specific alterations in midbrain connectivity. However, these results should be taken with a grain of salt and should be interpreted with care as they suffer from several limitations. First, they are based on a relatively small sample of subjects and patients and this may inherently reduce the statistical power of the analysis. Furthermore, they have been obtained from legacy data that do not correspond to modern standards for what concerns data acquisition; in particular, this work relies on low quality diffusion data (single shell datasets with low resolution, directions and b-values, no reverse phase encoding available); this features may have an influence on tractography results, despite they show a relatively good agreement with a previous work employing high quality data [25]. Due to the heterogeneous nature of the DWI preprocessing pipeline, we carefully inspected each output, in particular to ensure the good quality of non-linear registrations. In addition, we only evaluated FA from tractograms passing through SNc/VTA parcels, and we did not evaluated FA from specific targeted brain pathway (e.g., between SNc/VTA and different cortical regions) or adopting a voxel-wise approach. Finally, despite its popularity, the anatomical-pathological underpinnings of FA as a measure of white matter integrity are still far from being understood [83,84].

## 5. Conclusions

In the present work, we described a tripartite parcellation of dopaminergic midbrain regions according to their structural connectivity to striatal territories. We found that this topographical organization is maintained in both HC and SZ patients with no significant differences. We suggest that the midbrain parcellation scheme proposed herein may be helpful to achieve a more effective characterization of structural and functional anomalies of the dopaminergic midbrain in SZ patients. We hope that the present work may act as a trigger for further research leading to a better understanding of the complex pathophysiology of SZ.

## Figures and Tables

**Table 1:** Demographic and clinical features of the sample.

	HC		SZ	
	Mean	StD	Mean	StD
Age	31.75	11.76	32.56	10.50
Males	14	/	22	/
Females	10	/	8	/
Handedness (R/L/Both)	23/1/0	/	26/3/1	/
Mean Illness Duration (years)	/	/	9.26	8.73
Extrapyramidal Signs (SAS)	/	/	2.39	2.79
Negative Symptoms (SANS)	/	/	7.21	2.81
Positive Symptoms (SAPS)	/	/	4.82	2.25
Disorganized symptoms (SAPS)	/	/	2.04	2.23

StD: standard deviation. R: right. L: left. SAS: Simpson and Angus Scale; SANS: Scale for Assessment of Negative Symptoms; SAPS: Scale for Assessment of Positive Symptoms.

**Table 2:** Mean values for striatal and SNc/VTA streamline density index (SDI) and fractional anisotropy (FA) from SNc/VTA parcel connectivity analysis. StD: standard deviation.

			Striatal SDI		SNc/VTA SDI		FA	
			Mean	StD	Mean	StD	Mean	StD
Limbic	HC	Left	32.020	3.83	23.922	9.999	0.467	0.050
		Right	39.671	3.39	27.298	7.225	0.468	0.058
	SZ	Left	30.902	3.24	22.016	8.494	0.438	0.139
		Right	38.903	2.03	26.889	10.791	0.436	0.146
Prefrontal	HC	Left	24.725	1.95	26.050	11.162	0.474	0.047
		Right	32.462	2.9	23.838	8.271	0.436	0.146
	SZ	Left	25.503	2.03	29.570	7.817	0.488	0.061
		Right	32.78	3.21	28.228	12.131	0.440	0.144
Sensorimotor	HC	Left	34.171	3.71	44.516	11.582	0.477	0.045
		Right	30.121	2.22	41.457	12.972	0.487	0.050
	SZ	Left	31.932	3.68	43.388	9.056	0.445	0.140
		Right	31.131	2.25	36.982	11.084	0.447	0.149
